# RGB camera-based simultaneous measurements of percutaneous arterial oxygen saturation, tissue oxygen saturation, pulse rate, and respiratory rate

**DOI:** 10.3389/fphys.2022.933397

**Published:** 2022-09-19

**Authors:** Izumi Nishidate, Riku Yasui, Nodoka Nagao, Haruta Suzuki, Yohei Takara, Kaoru Ohashi, Fuminori Ando, Naoki Noro, Yasuaki Kokubo

**Affiliations:** ^1^ Tokyo University of Agriculture and Technology, Graduate School of Bio-Applications and Systems Engineering, Tokyo, Japan; ^2^ EBA Japan Co., Ltd., Tokyo, Japan; ^3^ Department of Neurosurgery, Faculty of Medicine, Yamagata University, Yamagata, Japan

**Keywords:** percutaneous arterial oxygen saturation, tissue oxygen saturation, pulse rate, respiratory rate, RGB camera

## Abstract

We propose a method to perform simultaneous measurements of percutaneous arterial oxygen saturation (*SpO*_2_), tissue oxygen saturation (*StO*_2_), pulse rate (*PR*), and respiratory rate (*RR*) in real-time, using a digital red–green–blue (RGB) camera. Concentrations of oxygenated hemoglobin (*C*_HbO_), deoxygenated hemoglobin (*C*_HbR_), total hemoglobin (*C*_HbT_), and *StO*_2_ were estimated from videos of the human face using a method based on a tissue-like light transport model of the skin. The photoplethysmogram (PPG) signals are extracted from the temporal fluctuations in *C*_HbO_, *C*_HbR_, and *C*_HbT_ using a finite impulse response (FIR) filter (low and high cut-off frequencies of 0.7 and 3 Hz, respectively). The *PR* is calculated from the PPG signal for *C*_HbT_. The ratio of pulse wave amplitude for *C*_HbO_ and that for *C*_HbR_ are associated with the reference value of *SpO*_2_ measured by a commercially available pulse oximeter, which provides an empirical formula to estimate *SpO*_2_ from videos. The respiration-dependent oscillation in *C*_HbT_ was extracted from another FIR filter (low and high cut-off frequencies of 0.05 and 0.5 Hz, respectively) and used to calculate the *RR*. *In vivo* experiments with human volunteers while varying the fraction of inspired oxygen were performed to evaluate the comparability of the proposed method with commercially available devices. The Bland–Altman analysis showed that the mean bias for *PR*, *RR*, *SpO*_2_, and *StO*_2_ were -1.4 (bpm), -1.2(rpm), 0.5 (%), and -3.0 (%), respectively. The precisions for *PR*, *RR*, *Sp O*_2_, and *StO*_2_ were ±3.1 (bpm), ±3.5 (rpm), ±4.3 (%), and ±4.8 (%), respectively. The resulting precision and RMSE for *StO*_2_ were pretty close to the clinical accuracy requirement. The accuracy of the *RR* is considered a little less accurate than clinical requirements. This is the first demonstration of a low-cost RGB camera-based method for contactless simultaneous measurements of the heart rate, percutaneous arterial oxygen saturation, and tissue oxygen saturation in real-time.

## Introduction

Quantitative evaluation of biological chromophores is useful for monitoring vital signs and physiological conditions. The major biological chromophores in skin tissues are melanin, oxygenated hemoglobin (HbO), and deoxygenated hemoglobin (HbR), which have unique optical absorption spectra in the visible to near-infrared wavelength range (Tuchin, 2007). When the concentrations of HbO and HbR vary, the corresponding change may be observed on a diffusely reflectance spectrum. Peripheral tissues demand a continuous supply of oxygen, which is delivered through blood circulation. The light absorption spectrum of hemoglobin is changed by the binding of oxygen to hemoglobin (Prahl, 2022). Delivery of oxygen to peripheral tissues can be deduced from the spectral diffuse reflectance by comparing it with the absorption spectra of HbO and HbR. The percentage of HbO in a volume of tissue is evaluated as tissue oxygen saturation (*StO*_2_) (Boas and Franceschini, 2011) and can be used as an indicator for monitoring oxygen consumption and hypoperfusion in peripheral tissues and cyanosis. The diffuse reflectance and transmittance spectra of peripheral tissue are changed by the periodic temporal variation in blood volume due to the cardiac pulse traveling through the body. Photoplethysmography (PPG) has been widely used to assess systemic physiological parameters such as heart rate, blood pressure, cardiac output, vascular compliance, and percutaneous oxygen saturation (*SpO*_2_) (Kamal et al., 1989; Allen, 2007).

Recent advances in imaging technology and computational methods have enabled imaging-based monitoring of vital signs. Camera-based vital sensing can remove the need for uncomfortable contact sensors. Moreover, they provide spatio-temporal information on the peripheral hemodynamic signals with only one sensor. The use of contactless devices could eliminate the risk of infectious diseases caused by contact sensing. In the last few years, due to the COVID-19 pandemic, it has become increasingly necessary to measure *SpO*_2_ and *RR* with a contactless device. The pulse wave observed in peripheral tissues is the most widely studied vital sign that has been evaluated by camera-based imaging photoplethysmography (iPPG) (Takano and Ohta, 2007; Verkruysse et al., 2008; Poh et al., 2010a; Poh et al., 2010b; Sun et al., 2011). Most RGB camera-based iPPG for *PR* detection evaluate the periodic changes in raw signals of the red, green, and/or blue color channels, without the quantification of hemoglobin. A wide variety of approaches and applications of iPPG have been proposed and can be viewed in the existing literature (McDuff et al., 2015; Rouast et al., 2018; Chen et al., 2019). Various techniques have been developed to extract a respiratory signal from a video sequence with iPPG (Mirmohamadsadeghi et al., 2016). Several methods have been used for evaluating *StO*_2_ in human skin tissue using an RGB camera (Tsumura et al., 2001; O’Doherty et al., 2009; He and Wang, 2020). Researchers have explored different approaches to extract *SpO*_2_ from peripheral blood circulation using a camera with visible and near-infrared light (Humphreys et al., 2005; Wieringa et al., 2005; Humphreys et al., 2007; Guazzi et al., 2015; Verkruysse et al., 2017).

Our research group has developed a simple and affordable imaging technique to evaluate transcutaneously multiple physiological parameters by using a digital red–green–blue camera (Nishidate et al., 2008; Nishidate et al., 2011). In this method, the *RGB* values were converted into tristimulus values in the Commission Internationale de l’Eclairage (CIE) XYZ color space, which is compatible with the common RGB color spaces. Monte Carlo simulation for light transport in biological tissue was then performed to specify the relationship between the *XYZ* values and the concentrations of oxygenated hemoglobin, deoxygenated hemoglobin, bilirubin, and melanin. The concentrations of total hemoglobin (*C*_HbT_) and tissue oxygen saturation (*StO*_2_) were also calculated from the estimated concentrations of oxygenated and deoxygenated hemoglobin. Based on this imaging method, a camera-based contactless measurement of *PR*, *StO*_2_, and *SpO*_2_ has been demonstrated through experiments with rats (Nishidate et al., 2020).

In this study, we further extend the method to perform simultaneous measurements of *SpO*_2_, *StO*_2_, pulse rate (*PR*), and respiratory rate (*RR*) in real-time. The pulse signals for *C*_HbO_, *C*_HbR_, and *C*_HbT_ are extracted by an FIR filter (low and high cut-off frequencies of 0.7 and 3 Hz, respectively). The value of the *HR* is calculated from the filtered *C*_HbT_ signal. The ratio of *PA*_HbO_ and *PA*_HbR_ is associated with the reference value of *SpO*_2_ measured by a commercially available pulse oximeter, which provides an empirical formula to estimate *SpO*_2_ from the PPG signals for *C*_HbO_ and *C*_HbR_. The value of the *RR* is calculated from the filtered signal for *C*_HbT_ extracted from another FIR filter (low and high cut-off frequencies of 0.05 and 0.5 Hz, respectively). The aim of this study is to evaluate the comparability of the proposed method with commercially available devices for monitoring *PR*, *RR*, *SpO*_2_, and *StO*_2_ in humans.

## Materials and methods

### Imaging system

A white-light-emitting diode (LED) (LA-HDF158, Hayashi Watch Works Co., Ltd., Tokyo, Japan) illuminated the facial skin of volunteers via a ring-shaped illuminator with a light guide (LGC1-8L1000-R55, Hayashi Watch Works Co., Ltd., Tokyo, Japan). The diffusely reflected light is received by a 24-bit RGB charge-coupled device camera (DFK23U618, Imaging source LLC, Charlotte, NC, United States) *via* an analyzer and a camera lens (FL-CC3516-2M, Ricoh Company, Ltd., Tokyo, Japan) to acquire an RGB color video at a resolution of 640 × 480 pixels. The ring-shaped polarizer mounted on the ring-shaped illuminator and analyzer was placed in a crossed Nicols alignment to reduce the specular reflection of light from the skin surface. A standard white diffuser with 99% reflectance (SRS-99-020, Labsphere Incorporated, New Hampshire, United States) was used to ensure the white balance of the camera and to correct the spatial nonuniformity of illumination. The facial color video of each volunteer was acquired at a sampling (frame) rate of 15 Hz. The illumination powers at 450, 520, and 632 nm on a subject’s skin were 46.13, 7.87, and 4.17 μW, respectively. The head of the subject was fixed on a head support (SR-HDR, SR Research Ltd., Ottawa, Canada) so that the distance between the face and the camera was constant. The working distance from the illuminator to a subject’s skin was 32 cm whereas that from the camera lens to a subject’s skin was 34 cm.

### Estimation of chromophore concentrations

We process the video recorded by the RGB imaging system based on a skin tissue model (Nishidate et al., 2008; Nishidate et al., 2011; Nishidate et al., 2020). First, the responses of the RGB channels in each pixel of the image are transformed into *XYZ* values with a matrix **M**_**1**_ as

$$\left[\begin{array}{l} X\\ Y\\ Z \end{array}\right]=M_{1}\left[\begin{array}{l} 1\\ R\\ G\\ B \end{array}\right],$$ (1)

where

$$M_{1}=\left[\begin{array}{l} \alpha_{0}\\ \beta_{0}\\ \gamma_{0} \end{array}\begin{array}{l} \alpha_{1}\\ \beta_{1}\\ \gamma_{1} \end{array}\begin{array}{l} \alpha_{2}\\ \beta_{2}\\ \gamma_{2} \end{array}\begin{array}{l} \alpha_{3}\\ \beta_{3}\\ \gamma_{3} \end{array}\right].$$ (2)

We experimentally determined the coefficients *α*_*i*_, *β*_*i*_, and *γ*i, (*i* = 0, 1, 2, 3) in Eq. 2 based on the measurements of a color checker (Color Checker, X-Rite Incorporated, Michigan, United States) that has 24 color standard patches and is supplied with datasets giving the *XYZ* values for each patch under specific illuminations and corresponding diffuse reflectance spectra. The calculated values of *X*, *Y*, and *Z* are then transformed into *C*_m_, *C*_HbO_, and *C*_HbR_ by matrix **M**_**2**_. To establish **M**_**2**_, 300 diffuse reflectance spectra in a wavelength range from 400–700 nm at intervals of 10 nm were numerically derived by Monte Carlo simulation (MCS) for light transport (Wang et al., 1995) in skin tissue under various values of *C*_m_, *C*_HbO_, and *C*_HbR_ and, then obtained the corresponding *XYZ* values. A more detailed description of the skin tissue model including optical parameters can be found elsewhere (Nishidate et al., 2011; Nishidate et al., 2020). Multiple regression analysis with 300 datasets established three multiple regression equations as empirical formulae for *C*_m_, *C*_HbO_, and *C*_HbR_:

$$C_{m}=\eta_{0}+\eta_{1}X+\eta_{2}Y+\eta_{3}Z+\eta_{4}X^{2}+\eta_{5}Y^{2}+\eta_{6}Z^{2}+\eta_{7}XY+\eta_{8}XZ+\eta_{9}YZ,$$ (3)

$$C_{HbO}=\rho_{0}+\rho_{1}X+\rho_{2}Y+\rho_{3}Z+\rho_{4}X^{2}+\rho_{5}Y^{2}+\rho_{6}Z^{2}+\rho_{7}XY+\rho_{8}XZ+\rho_{9}YZ,$$ (4)

$$C_{HbR}=\sigma_{0}+\sigma_{1}X+\sigma_{2}Y+\sigma_{3}Z+\sigma_{4}X^{2}+\sigma_{5}Y^{2}+\sigma_{6}Z^{2}+\sigma_{7}XY+\sigma_{8}XZ+\sigma_{9}YZ.$$ (5)

The estimations of *C*_m_, *C*_HbO_, and *C*_HbR_ obtained from the empirical formulae with only the first-order terms of *XYZ* values have been validated in a prior work (Nishidate et al., 2011; Nishidate et al., 2020). Although we leverage the approach proposed in the prior work (Nishidate et al., 2011; Nishidate et al., 2020) to estimate *C*_m_, *C*_HbO_, and *C*_HbR_, we newly include the second-order terms of *XYZ* values into predictor variables in the multiple regression model to improve the accuracies of *C*_m_, *C*_HbO_, and *C*_HbR_. The multiple regression coefficients *η*_*i*_, *ρ*_*i*_, and *σ*_*i*_, (*i* = 0, 1, 2, ..., 9) in Eqs 3–5 represent the contributions of the *XYZ* values to *C*_m_, *C*_HbO_, *C*_HbR_, and *C*_bil_, respectively, and are used as the elements of matrix **M**_**2**_ as

$$M_{2}=\left[\begin{array}{l} \eta_{0}\\ \rho_{0}\\ \sigma_{0} \end{array}\begin{array}{l} \eta_{1}\\ \rho_{1}\\ \sigma_{1} \end{array}\begin{array}{l} \eta_{2}\\ \rho_{2}\\ \sigma_{2} \end{array}\begin{array}{l} \eta_{3}\\ \rho_{3}\\ \sigma_{3} \end{array}\begin{array}{l} \eta_{4}\\ \rho_{4}\\ \sigma_{4} \end{array}\begin{array}{l} \eta_{5}\\ \rho_{5}\\ \sigma_{5} \end{array}\begin{array}{l} \eta_{6}\\ \rho_{6}\\ \sigma_{6} \end{array}\begin{array}{l} \eta_{7}\\ \rho_{7}\\ \sigma_{7} \end{array}\begin{array}{l} \eta_{8}\\ \rho_{8}\\ \sigma_{8} \end{array}\begin{array}{l} \eta_{9}\\ \rho_{9}\\ \sigma_{9} \end{array}\right].$$ (6)

The transformation with **M**_**2**_ from the *XYZ* values to concentrations of melanin, oxygenated hemoglobin, and deoxygenated hemoglobin is, thus, expressed as

$$\left[\begin{array}{l} C_{m}\\ C_{HbO}\\ C_{HbR} \end{array}\right]=M_{2}\left[\begin{array}{l} 1\\ X\\ Y\\ Z\\ X^{2}\\ Y^{2}\\ Z^{2}\\ XY\\ XZ\\ YZ \end{array}\right].$$ (7)

To confirm the effect of the second-order terms of *XYZ* on the accuracy of the estimated concentrations, we evaluate the coefficient of determination *R* ^2^ of the regression models. The values of *R* ^2^ for *C*_m_, *C*_HbO_, and *C*_HbR_ in the regression model with only the first-order terms of *XYZ* values were 0.88, 0.81, and 0.70, respectively, whereas those in the regression model with the first- and second-order terms were 0.99, 0.98, and 0.93, respectively. Once we establish the matrices **M**_**1**_ and **M**_**2**_, images of *C*_m_, *C*_HbO_, and *C*_HbR_ are reconstructed from a digital RGB image without the MCS. The total hemoglobin concentration is simply calculated as *C*_HbT_ = *C*_HbO_ + *C*_HbR_ and tissue oxygen saturation of hemoglobin as *StO*_2_% = (*C*_HbO_/*C*_HbT_) × 100.

### Calculation of percutaneous arterial oxygen saturation, tissue oxygen saturation, heart rate, and respiratory rate

**FIGURE 1:**
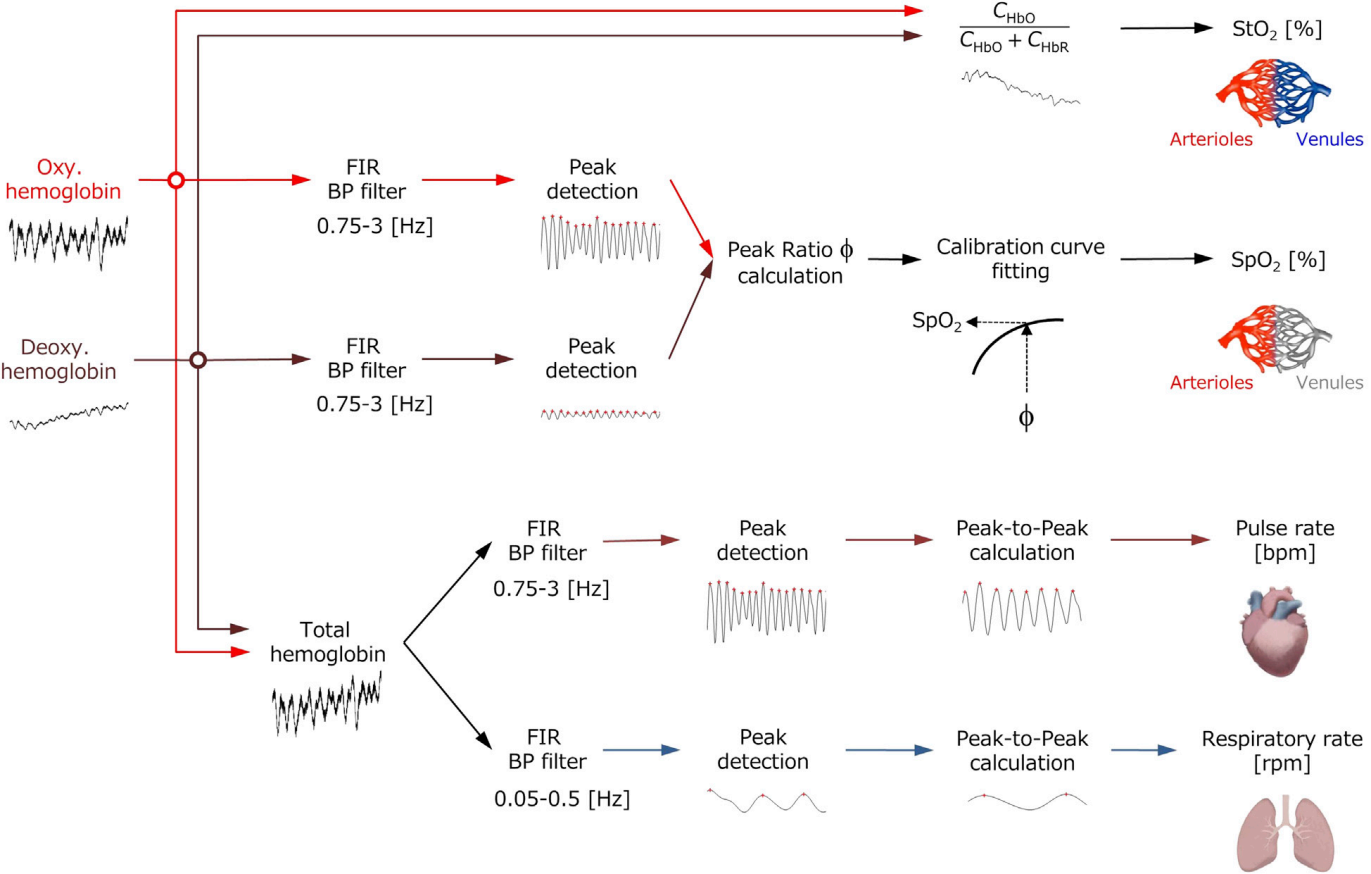
Flowchart of the process for extracting the values of pulse rate *PR*, respiratory rate *RR*, percutaneous arterial oxygen saturation *SpO*_2_, and tissue oxygen saturation *StO*_2_ from the time courses of oxygenated hemoglobin concentration *C*_HbO_, deoxygenated hemoglobin concentration *C*_HbR_, and total hemoglobin concentration *C*_HbT_.

Figure 1 shows a flowchart describing the estimation process using the proposed method. In our previous works (Nishidate et al., 2008; Nishidate et al., 2011; Nishidate et al., 2020), we performed all the analysis and processing to calculate the physiological parameters offline, but in this study, we have newly developed a system that can process and display them online in real-time. Also, we added the calculation of *RR* to the system developed in this study although in the previous study (Nishidate et al., 2020) we did not consider the *RR*. The PPG signals for *C*_HbO_, *C*_HbR_, and *C*_HbT_ are extracted by an FIR filter (low and high cut-off frequencies of 0.75 and 3 Hz, respectively). The value of the *PR* is calculated from the filtered *C*_HbT_ signal. The value of the *RR* is calculated from the filtered signal for *C*_HbT_ extracted from another FIR filter (low and high cut-off frequencies of 0.05 and 0.5 Hz, respectively). We assume that the pulse wave amplitude for *C*_HbO_ (*PA*_HbO_) and for *C*_HbR_ (*PA*_HbR_) is decreased and increased as *SpO*_2_ is decreased. The ratio of *PA*_HbO_ and *PA*_HbR_ is calculated as

$$\phi =\frac{{PA}_{HbO}}{{PA}_{HbR}}.$$ (8)

**FIGURE 2:**
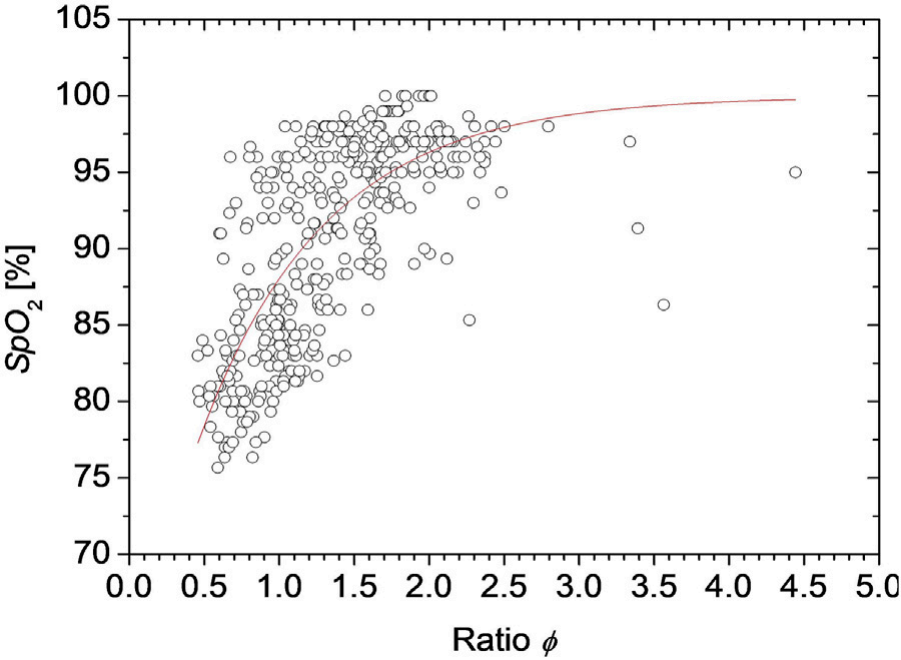
Dependence of *SpO*_2_ measured by the pulse oximeter on the value of *ϕ* obtained from the proposed method.

The value of *ϕ* is associated with the reference value of *SpO*_2_ measured by a commercially available pulse oximeter, which provides an empirical formula to estimate *SpO*_2_ from the PPG signals for *C*_HbO_ and *C*_HbR_. Figure 2 shows the dependence of *SpO*_2_ measured by the pulse oximeter on the value of *ϕ* obtained from the proposed method. Once the value of *ϕ* reaches 3.0, the curve is almost flat, indicating there is little change in *SpO*_2_ above this point. But, at less than 2.0 of *ϕ*, the curve is very steep, and small changes in *ϕ* greatly reduce *SpO*_2_. We approximated the function *SpO*_2_ (*ϕ*) as an exponential function as

$$SpO_{2}\left(\phi \right)=Aexp\left(-\frac{\phi }{B}\right)+C.$$ (9)

The estimated value of SpO_2_ was obtained from the value of *ϕ* extracted from the iPPG signals for *C*_HbO_ and *C*_HbR_ through Eq. 8. *StO*_2_ is directly calculated from *C*_HbO_ and *C*_HbR_ as described in *Estimation of chromophore concentrations*. We integrated the aforementioned process using Matlab/simlink^®^ and created a real-time monitoring system for *PR*, *RR*, *SpO*_2_, and *StO*_2_.

### Experimental protocols

**FIGURE 3:**
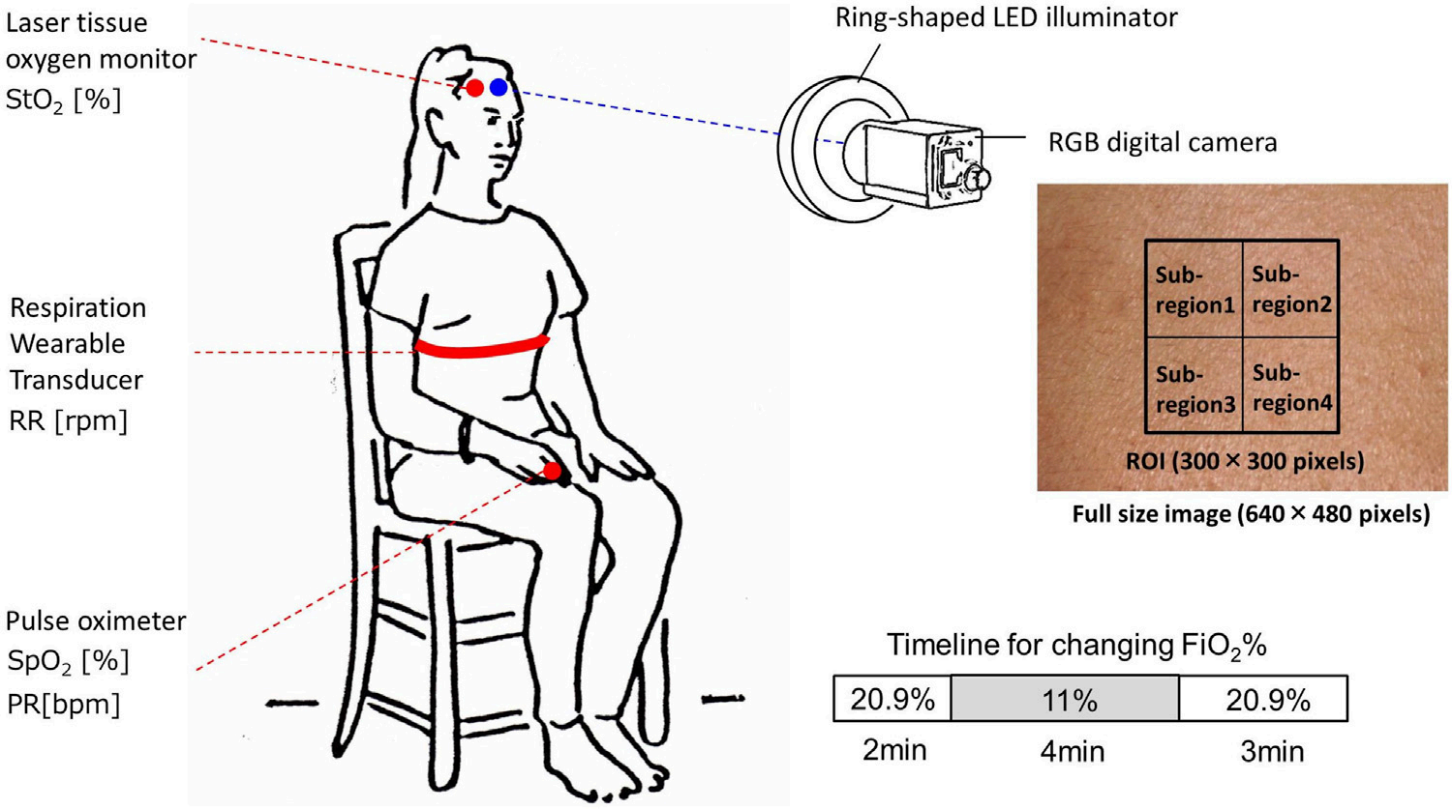
Schematic illustration of the experimental setup used for data collection while changing inspired oxygen.

Figure 3 shows the experimental setup used in this study. Our experiment features 13 healthy volunteers of both genders of different ages (22–45 years). Informed consent was obtained from all participants prior to the data being collected. During the experiment, for 9 min, participants were seated and the temperature of the room was held constant. Each volunteer was exposed to normoxia and hypoxia by inhaling the mixture of O_2_ and N_2_, with the fraction of inspired oxygen (FiO_2_) being 11% for 4 min, for which a breath mask connected with the Douglas bag was used under spontaneous respiration. The value of FiO_2_ was monitored by using an oxygen gas sensor (OM-25MS10; TAIEI DENKI, Tokyo, Japan). This protocol was approved by the institutional review board of the Tokyo University of Agriculture and Technology (approval numbers 200907-0241 and 210903-0338) and all methods were performed in accordance with the protocol. Simultaneously with optical imaging for skin tissue, *SpO*_2_ and *PR* were measured by a pulse oximeter (OxyTrue^®^ A SMARTsat, bluepoint MEDICAL GmbH&Co.KG; Selmsdorf, Germany) with a finger sensor. The *RR* was measured by using a respiration wearable transducer (BIONOMADIX^®^; Biopac Systems Inc., Goleta, CA, United States). *StO*_2_ was measured by a laser tissue oxygen monitor (BOM-L1TRSF; OMEGAWAVE, Inc., Tokyo, Japan).

### Statistical considerations

We used only one area of the forehead of each volunteer for data analysis of the RGB video. Region of interest (ROI) of 300 × 300 pixels corresponding to the area of 1.76 × 1.76 cm^2^ was set in each video frame and the mean and the standard deviation (SD) over the ROI were calculated for the analysis of time courses in *C*_HbO_, *C*_HbR_, *C*_HbT_, and *StO*_2_. Therefore, data are expressed as mean ± SD. The ROI was divided into four sub-regions to evaluate the spatial variability of each physiological parameter in the ROI as shown in Figure 3. The root mean squared error (RMSE) and Pearson’s correlation coefficients were calculated using the ground truth values measured by the commercially available devices and estimated values by the proposed method. A probability value of *p* < 0.05 indicates statistical significance. To investigate the agreement between the estimated values obtained by the proposed method and the ground truth values, we performed a Bland–Altman (BA) analysis. In the BA analysis, a BA diagram with the horizontal axis representing the mean of the estimated values obtained by the proposed method and the ground truth values and the vertical axis representing the difference between the estimated values obtained by the proposed method and the ground truth values is used to graphically represent the data, displaying mean bias, precision (mean bias±SD), and limits of agreement (mean bias±2SD).

## Results and discussion

**FIGURE 4:**
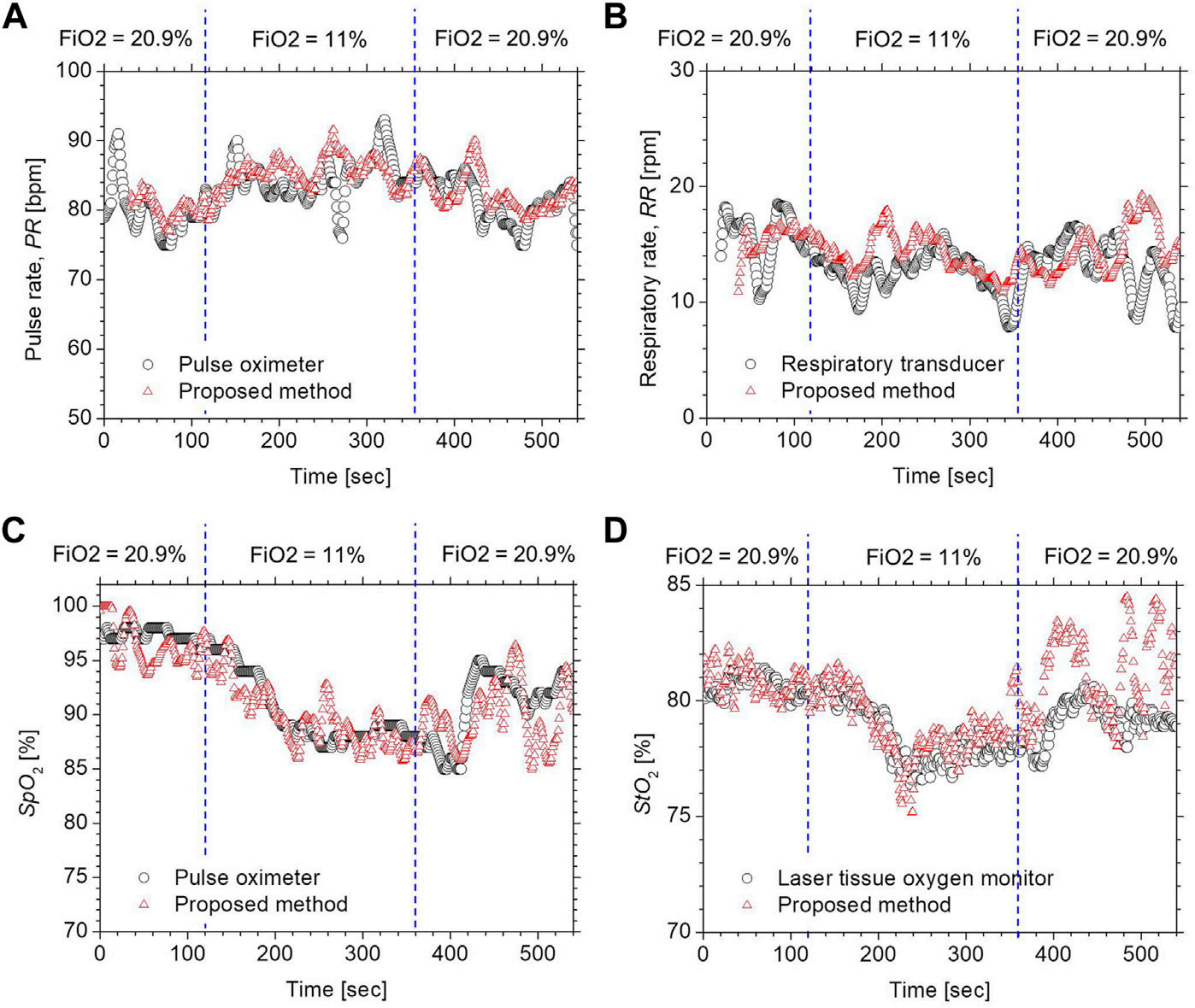
Typical time courses of **(A)** *PR*, **(B)** *RR*, **(C)** *Sp*O_2_, and **(D)** *StO*_2_ while changing inspired oxygen. The black solid line and red solid line in each figure represent the measured value by a commercially available device and the estimated value obtained by the proposed method.

Figure 4 compares the typical time courses of (a) *PR*, (b) *RR*, (c) *Sp*O_2_, and (d) *StO*_2_ between the estimated values by the proposed method and the ground truth values measured by the commercially available devices obtained from a volunteer while changing FiO_2_. The estimated values of *Sp*O_2_ and *StO*_2_ gradually decreased after the onset of hypoxia and fell to approximately 87 and 77%, respectively. After the end of hypoxia, *Sp*O_2_ and *StO*_2_ returned to their normal levels. Time courses of *Sp*O_2_ and *StO*_2_ while changing FiO_2_ were consistent with well-known physiological responses to changes in FiO_2_. The estimated value of the *PR* increased during hypoxia, which was indicative of compensating for hypoxia. The estimated value of the *RR* was slightly decreased during hypoxia, which is probably due to the decrease in the respiratory rate caused by an increase in tidal volume. Overall trends in *PR*, *RR*, *SpO*_2_, and *StO*_2_ estimated by the proposed method coincide with those measured by the commercially available devices.

**FIGURE 5:**
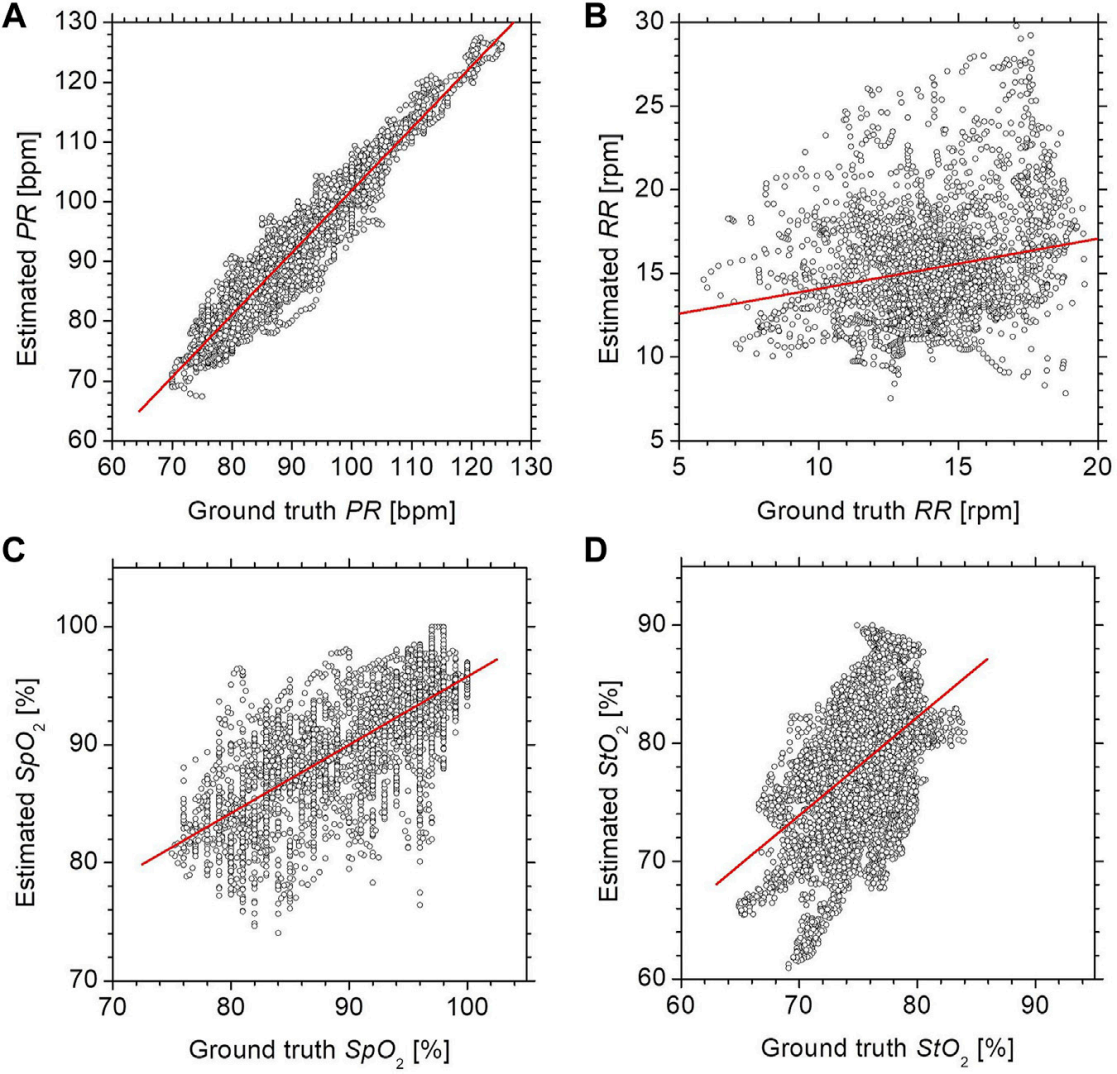
Scatter plots comparing the estimated values by the proposed method with the ground truth values measured by commercially available devices for **(A)** *PR*, **(B)** *RR*, **(C)** *SpO2*, and **(D)** *StO*_2_.

Figure 5 shows scatter plots comparing the estimated values by the proposed method with the ground truth values measured by commercially available devices for (a) *PR*, (b) *RR*, (c) *SpO2*, and (d) *StO*_2_. The correlation coefficients for *PR* and *SpO*_2_ were 0.96 (*p* < 0.0001) and 0.76 (*p* < 0.0001), respectively, indicating that those physiological parameters obtained by the proposed method are well-correlated with the measurements of existing devices. *StO*_2_ showed a moderate correlation between the two methods with a correlation coefficient of 0.46 (*p* < 0.0001). The correlation coefficient for *StO*_2_ was 0.46 (*p* < 0.0001), indicating that the measurements obtained by the proposed method were moderately correlated with those by the commercially available laser tissue oxygen monitor. On the other hand, the *RR* showed a low correlation between the two methods with a correlation coefficient of 0.22 (*p* < 0.0001).

**FIGURE 6:**
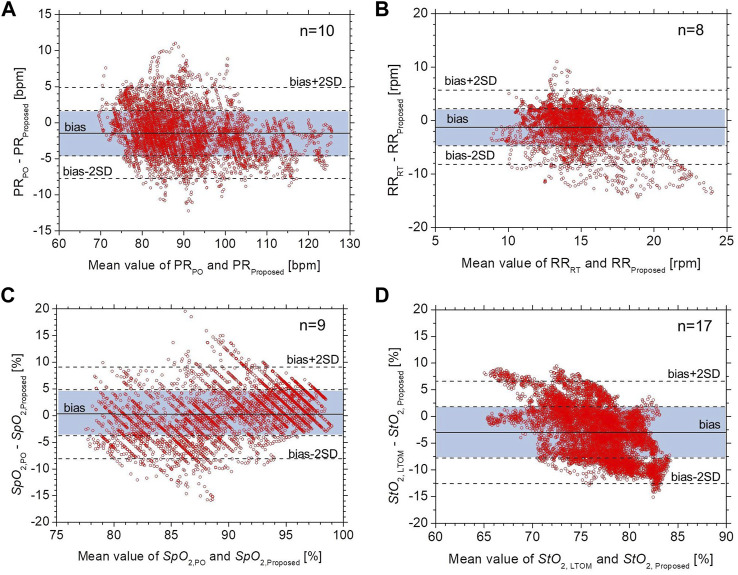
Bland–Altman plots for **(A)** *PR*, **(B)** *RR*, **(C)** *SpO*_2_, and **(D)** *StO*_2_. The black solid line represents the mean bias, black dotted lines represent the mean difference ± precision, and the dotted lines represent the accepted limits of agreement.

Figure 6 shows Bland–Altman plots for (a) *PR*, (b) *RR*, (c) *SpO*_2_, and (d) *StO*_2_. In Figure 6, the black solid line represents the mean bias (defined as the mean difference in measurements between the proposed method and commercially available devices), black dotted lines represent the mean difference ± precision (mean difference ±SD), and the dotted lines represent the accepted limits of agreement (mean difference ± 2SD). The values of mean bias for *PR* and *SpO*_2_ were -1.4 (bpm) and 0.5 (%), respectively. Limits of agreement ranged from -7.7–4.8 (bpm) and -8.1–9.1 (%) for *PR* and *SpO*_2_, respectively. The resulting precisions for *PR* and *SpO*_2_ were ±3.1 (bpm) and ±4.3 (%), respectively, whereas the RMSE for *PR* and *SpO*_2_ were 3.5 (%) and 4.3 (%), respectively. HR measurements were considered accurate if the mean absolute difference was within either ±10% or ±5 bpm (ANSI/AAMI, 2002) while *SpO*_2_ measurements were considered accurate if the mean RMSE was ≤3.0% (Center for Devices and Radiological Health Food and Drug Administration, 2013). The accuracy of *PR* and *SpO*_2_ is comparable with that required for the pulse oximeter devices. In contrast, the values of mean bias for *RR* and *StO*_2_ were -1.2 (rpm) and -3.0 (%), respectively. Limits of agreement ranged from -8.2–5.7 (rpm) and -12.6–6.6 (%) for *RR* and *StO*_2_, respectively. The resulting precisions for *RR* and *StO*_2_ were ±3.5 (rpm) and ±4.8 (%), respectively, whereas the RMSE for *RR* and *StO*_2_ were 3.7 (%) and 5.7 (%), respectively. The accuracy of the *RR* is considered a little less accurate than clinical requirements.

Our results suggest that the proposed RGB camera-based contactless method could provide equivalent precisions in *PR* and *SpO*_2_ to an existing pulse oximeter. However, the calculation of *SpO*_2_ depends on the optical path length difference between different wavelengths. Regular pulse oximeters with transillumination work due to the transmitted light from a finger being less variable than the diffusely reflected light in terms of wavelengths’ dependence on the optical path length. This could be a cause of deviations in *SpO*_2_ between the pulse oximeter and the proposed method as shown in Figure 5.

**FIGURE 7:**
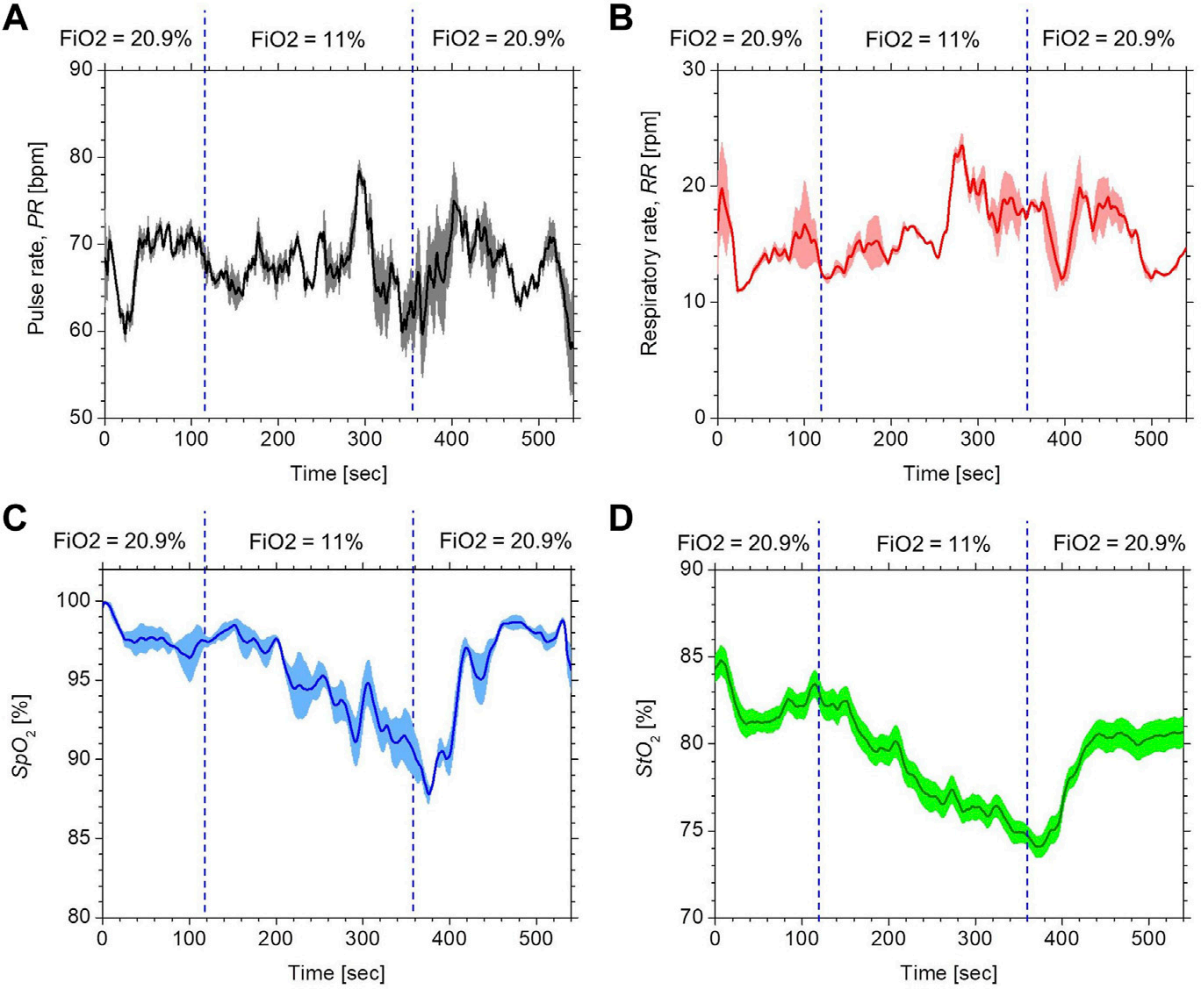
Typical time courses of the mean (bold line) ± standard deviation (shaded area) for **(A)** *PR*, **(B)** *RR*, **(C)** *SpO*_2_, and **(D)** *StO*_2_ over the four sub-regions of ROI obtained from a subject.

The resulting precision and RMSE for *StO*_2_ were pretty close to the clinical accuracy requirement. One possible explanation for the deviations in *StO*_2_ between the two instruments shown in Figure 5 may be because those are continuous wave instruments. The calculation of *StO*_2_ in the proposed method is an approximation and will probably be spatially variable across the ROI. We evaluated the spatial variability of each physiological parameter in the ROI. Figure 7 shows the typical time courses of the mean ± standard deviation of (a) *PR*, (b) *RR*, (c) *SpO*_2_, and (d) *StO*_2_ over the four sub-regions of ROI obtained from a subject. The average values of the standard deviation over the period for *PR*, *RR*, *SpO*_2_, and *StO*_2_ were 1.77 bpm, 0.98 rpm, 0.81%, and 0.75%, respectively. Moderate correlation with the existing device for *StO*_2_ may also be due to the difference in the probing depth between the proposed method and the laser tissue oxygen monitor. The laser tissue oxygen monitor used in this study calculates the concentrations of oxygenated hemoglobin and deoxygenated hemoglobin from measurements of spatially resolved diffuse reflectance at 635, 655, and 690 nm, and its probing depth is approximately 3 mm when the source-detector separation is 3 mm. The average probing depth of the proposed method in which the responses of red, green, and blue channels were used is expected to be smaller than that of the laser tissue oxygen monitor. This may contribute to the discrepancy in *StO*_2_. The calculation of *StO*_2_ in the laser tissue oxygen monitor used as the ground truth is also an approximation and will probably be spatially variable. Generally speaking, a continuous wave instrument based on diffuse reflectance without spatial-resolved and/or frequency domain measurements obtains relative changes in absorption but not absolute value as it does not work for measuring light scattering coefficients. However, the possibility of relative measurements of *StO*_2_ could still prove valuable.

We used the skin tissue Monte Carlo simulation model with only one typical light scattering spectrum and a typical thickness for the epidermis and the dermis to establish the empirical formulae for estimating chromophore concentrations. However, the actual skin thickness and light scattering spectrum could vary with subjects and parts of the body. Changes in the light scattering properties of tissue and the thickness of each layer will impact the regression equations, and thus, the calculation of *StO*_2_. The skin tissue model with variations in the scattering spectrum and the thickness of each layer could improve the accuracy of *StO*_2_. Our results revealed that the current system needs to improve the accuracy of *RR* measurements. Using slow fluctuation in peripheral blood volume to measure the respiratory rate remains challenging. Our results of the *RR* showed the low precision and correlation coefficient between the proposed method and the respiration wearable transducer. Respiration-dependent fluctuation in *C*_HbT_ was weak compared with the plethysmogram for *C*_HbT_. This may have contributed to the lack of accuracy in peak detection for calculating *RR*.

There are several smartphone applications for monitoring both *PR* and *SpO*_2_ such as Oximeter (Ox, digiDoc technologies, Egersund, Norway) and Heart Rate & Pulse Oximeter (Pox, produced by LIJUN LIU). However, those applications are based on contact measurements of diffusely reflected light that require a subject to cover the flash and camera lens with a fingertip. Moreover, the range of *SpO*_2_ is 93–100% in each application. As a non-contact device for *SpO*_2_, Verkruysse et al. reported a camera-based iPPG technique (Verkruysse et al., 2017). This is the first study that has been able to demonstrate that calibrated non-contact monitoring of *SpO*_2_, *PR*, and *RR* is possible. This technique is based on multispectral imaging using two tripod-mounted monochrome cameras with spectral bandpass filters with red and infrared (IR) center wavelengths of 675 and 842 nm, respectively, and not an RGB camera-based iPPG. Guazzi et al. developed a non-contact RGB-camera-based monitor method for *SpO*_2_ (Guazzi et al., 2015). This method calculates *SpO*_2_ and *PR* by analyzing the time series of raw signals for each of the R, G, and B channels. However, it cannot monitor *StO*_2_ since *C*_HbO_ and *C*_HbR_ are not estimated from the *RGB* values in this model. Therefore, our proposed method is unique and novel, different from any other smartphone applications or iPPG techniques mentioned previously.

In this study, we set the frequency bands of FIR filters as 0.75–3 Hz for the PPG signals and 0.05–0.5 Hz for the respiratory signal. These ranges correspond to pulse rates of 45–180 bpm and respiratory rates of 3–30 rpm. However, they do not necessarily include physiological extremes relevant in a medical setting. For example, in patients with atrioventricular block, the pulse rate could be ranged from 30–40 bpm. To detect such a slow heart rate, the low cut-off frequency of the FIR filter for the PPG signal should be set to 0.5 or less. In such a case, the frequency bands for the pulse rate and respiratory rate will partially overlap. As a result, it will be difficult to distinguish between pulse and respiratory signals. We took the variation in melanin concentration into account for the Monte Carlo model to establish the empirical formulae for *C*_HbO_ and *C*_HbR_. However, if the actual melanin concentration is out of a setting range in the Monte Carlo model (e.g., subjects with darker skin tones), the estimation errors in *C*_HbO_ and *C*_HbR_ could be increased. Moreover, it will become difficult to detect temporal fluctuations of *C*_HbO_, *C*_HbR_, and *C*_HbT_ due to the reduced diffuse reflection caused by the higher melanin concentration. In our experiments, the working distance of the camera, lighting, and body motions were carefully controlled while collecting data. Under real-world situations, the measurement is likely to be more challenging as the conditions could be variable and may not be strictly controlled. Using a real-time face detection and tracking technique with automatic exposure and white balance control in the developed system will be useful to reduce motion artifacts and prevent accuracy deterioration in each vital sign estimation. Although we evaluate peripheral hemodynamics in forehead skin to extract the four vital parameters, our method can be applied to the other parts of the body surface. The accuracy of the measurements of *PR*, *RR*, *SpO*_2_, and *StO*_2_ for different skin regions should be investigated in the future.

## Conclusion

In summary, a method to perform simultaneous measurements of percutaneous arterial oxygen saturation, tissue oxygen saturation, pulse rate, and respiratory rate in real-time using an RGB camera was demonstrated in the present study. *In vivo* experiments with human volunteers while varying the fraction of inspired oxygen were performed to evaluate the comparability of the proposed method with commercially available devices. The results of correlation analysis and Bland–Altman plots showed that the proposed RGB camera-based contactless method could provide equivalent precisions in *PR* and *SpO*_2_ to an existing pulse oximeter. The resulting precision in *StO*_2_ was pretty close to the accuracy requirement. The current system needs to improve the accuracy of *RR* measurement. To the best of our knowledge, this is the first demonstration of simultaneous contactless measurements of pulse rate, percutaneous arterial oxygen saturation, and tissue oxygen saturation in human subjects using a digital RGB camera. The results in this study indicate the potential of this method for remote monitoring multiple physiological parameters and vital signs that enable cost-effective, easy-to-use, contactless, and point-of-care devices.

## Data Availability

The original contributions presented in the study are included in the article/Supplementary Material; further inquiries can be directed to the corresponding author.
